# Supplementary material for: Effect of aging and body characteristics on facial sexual dimorphism in the Caucasian Population
Source: PLoS One. 2020 May 14;15(5):e0231983. doi: 10.1371/journal.pone.0231983 (PMC7224454; doi:10.1371/journal.pone.0231983)
Supplement: Attachment — Submitted filename: 1 RESPONSE TO REVIEWERS.docx [file pone.0231983.s004.docx]

Dear Dr. Mateusz Koziej,

Thank you for considering our article entitled “Effect of aging and body characteristics on facial sexual dimorphism in the Caucasian population” in PLOS ONE. We believe that our article makes an important contribution to the understanding of the facial differences between men and women and the impact of aging.

We appreciate the editors’ comments, and they have all been incorporated into the revised manuscript. Below are the answers to the editors’ comments and suggestions. Changes in the revised manuscript are highlighted in yellow.

The manuscript was edited by Dr Paul McGuiness, a professional proofreader and a native speaker of English.

All the figures have been improved and corrected with the PACE digital diagnostic tool to meet the PLOS requirements for figure quality and format.

Yours sincerely,

Zala Skomina, DMD

**Our Answers to Editor:**

*In table 1. Please provide quartiles Q1,Q3.*

Quartiles Q1, Q3 were added in Table 1.

*Material – change for “Study group”*

Material section was changed in Study group.

*Methods – change for Protocol*

Methods section was changed in Protocol section.

*I would like to ask authors to include in statistical analysis section 1-2 sentences regarding how to interpret the MLR test. Just to make it clear for the reader. What significant coefficient in this analysis actually means.*

Based on this comment we now provide a better interpretation of the multiple-linear-regression model and the meaning of signifiant coefficient in the Statistical analysis section:
The significant regression coefficient (marked with *; **; *** in Table 3) shows you how much the dependent variable is expected to change when that independent variable increases by one, holding all the other independent variables constant. For example; in older group face width is 7 mm longer than in younger group with unchanged BMI, body height and gender.
